# Early, precise, and safe clinical evaluation of the pharmacodynamic effects of novel agents in the intact human tumor microenvironment

**DOI:** 10.3389/fphar.2024.1367581

**Published:** 2024-04-12

**Authors:** Kenneth R. Gundle, Karthik Rajasekaran, Jeffrey Houlton, Gary B. Deutsch, Thomas J. Ow, Robert G. Maki, John Pang, Cherie-Ann O. Nathan, Daniel Clayburgh, Jason G. Newman, Elyse Brinkmann, Michael J. Wagner, Seth M. Pollack, Matthew J. Thompson, Ryan J. Li, Vikas Mehta, Bradley A. Schiff, Barry I. Wenig, Paul L. Swiecicki, Alice L. Tang, Jessica L. Davis, Annemieke van Zante, Jessica A. Bertout, Wendy Jenkins, Atticus Turner, Marc Grenley, Connor Burns, Jason P. Frazier, Angela Merrell, Kimberly H. W. Sottero, Jonathan M. J. Derry, Kate C. Gillespie, Bre Mills, Richard A. Klinghoffer

**Affiliations:** ^1^ Department of Orthopaedics and Rehabilitation, Oregon Health and Science University, Portland, OR, United States; ^2^ Portland Veterans Affairs Medical Center, Portland, OR, United States; ^3^ Department of Otorhinolaryngology—Head and Neck Surgery, University of Pennsylvania, Philadelphia, PA, United States; ^4^ Sarah Cannon Research Institute, Charleston, SC, United States; ^5^ Zucker School of Medicine at Hofstra/Northwell, New Hyde Park, NY, United States; ^6^ Department of Otorhinolaryngology-Head and Neck Surgery, Montefiore Medical Center/Albert Einstein College of Medicine, Bronx, NY, United States; ^7^ Department of Pathology, Montefiore Medical Center/Albert Einstein College of Medicine, Bronx, NY, United States; ^8^ Cold Spring Harbor Laboratory, Cold Spring Harbor, NY, United States; ^9^ Department of Otolaryngology/Head and Neck Surgery, Louisiana State University Health Shreveport, Shreveport, LA, United States; ^10^ Department of Otolaryngology‐Head and Neck Surgery, Oregon Health and Science University, Portland, OR, United States; ^11^ Department of Orthopaedics and Sports Medicine, University of Washington School of Medicine, Seattle, WA, United States; ^12^ Division of Oncology, University of Washington, Seattle, WA, United States; ^13^ Clinical Research Division, Fred Hutchinson Cancer Research Center, Seattle, WA, United States; ^14^ Department of Otolaryngology—Head and Neck Surgery, University of Illinois at Chicago, Chicago, IL, United States; ^15^ Department of Hematology Oncology, University of Michigan Medical School, Ann Arbor, MI, United States; ^16^ Department of Otolaryngology—Head and Neck Surgery, University of Cincinnati College of Medicine, Cincinnati, OH, United States; ^17^ Department of Pathology and Laboratory Medicine, Indiana University School of Medicine, Indianapolis, IN, United States; ^18^ Department of Pathology, University of California San Francisco, San Francisco, CA, United States; ^19^ Presage Biosciences, Inc., Seattle, WA, United States

**Keywords:** phase 0, intratumoral microdosing, spatial profiling, multidrug analyses, pharmacodynamics, tumor microenvironment, drug development

## Abstract

**Introduction:** Drug development is systemically inefficient. Research and development costs for novel therapeutics average hundreds of millions to billions of dollars, with the overall likelihood of approval estimated to be as low as 6.7% for oncology drugs. Over half of these failures are due to a lack of drug efficacy. This pervasive and repeated low rate of success exemplifies how preclinical models fail to adequately replicate the complexity and heterogeneity of human cancer. Therefore, new methods of evaluation, early in the development trajectory, are essential both to rule-in and rule-out novel agents with more rigor and speed, but also to spare clinical trial patients from the potentially toxic sequelae (high risk) of testing investigational agents that have a low likelihood of producing a response (low benefit).

**Methods:** The clinical *in vivo* oncology (CIVO^®^) platform was designed to change this drug development paradigm. CIVO precisely delivers microdose quantities of up to 8 drugs or combinations directly into patient tumors 4–96 h prior to planned surgical resection. Resected tissue is then analyzed for responses at each site of intratumoral drug exposure.

**Results:** To date, CIVO has been used safely in 6 clinical trials, including 68 subjects, with 5 investigational and 17 approved agents. Resected tissues were analyzed initially using immunohistochemistry and *in situ* hybridization assays (115 biomarkers). As technology advanced, the platform was paired with spatial biology analysis platforms, to successfully track anti-neoplastic and immune-modulating activity of the injected agents in the intact tumor microenvironment.

**Discussion:** Herein we provide a report of the use of CIVO technology in patients, a depiction of the robust analysis methods enabled by this platform, and a description of the operational and regulatory mechanisms used to deploy this approach in synergistic partnership with pharmaceutical partners. We further detail how use of the CIVO platform is a clinically safe and scientifically precise alternative or complement to preclinical efficacy modeling, with outputs that inform, streamline, and de-risk drug development.

## Introduction

Development costs to bring a safe and effective new drug to market span hundreds of millions to several billion dollars (Wouters et al., 2020a; Schuhmacher et al., 2023), with the overall likelihood of approval for all candidate assets estimated at 10% or less (Morgan et al., 2011; Hay et al., 2014; Smietana et al., 2016; Wong et al., 2019; Wouters et al., 2020a). Assets in development in the oncology space epitomize this problem and include both the highest development costs and lowest clinical success rates of any class of drugs (Wong et al., 2019; Wouters et al., 2020a). An estimated $50–60 billion are spent each year on oncology trials for novel therapeutics that ultimately fail (Jentzsch et al., 2023). The human patients who consent to participate in these trials bear the burden of a system that, overall, presents a highly imbalanced risk-benefit ratio. Innovative approaches are needed to improve the economics, precision, and ethics of drug development.

Retrospective analyses have found that including biomarkers for patient selection can double (or, in the case of oncology, sextuple) the likelihood of drug development success (Smietana et al., 2016; Wong et al., 2019). These gains have largely been attributed to the availability of large genomic and transcriptomic datasets enabling patient stratification according to baseline disease characteristics. Unfortunately, similar retrospective datasets are not available for early-phase investigational drugs, and biomarkers predicting drug toxicity or efficacy must instead be extrapolated from preclinical translational models. Even the most sophisticated preclinical models fail to replicate the diversity and complexity of the human tumor microenvironment (TME) which is composed of a heterogeneous and dynamic bionetwork of tumor cells, immune cells, endothelial cells, fibroblasts, and extracellular matrix, among other elements. These various components constantly interact in both negative and positive ways with significant impact on tumorigenesis, cancer progression, and most importantly, modulating responses to treatment and clinical outcomes (Di Modugno et al., 2019). As a result, traditional preclinical models cannot be used as a reliable proxy to inform stratification strategies for drugs in development (Khalil et al., 2020; Pan et al., 2020). Even more recently developed *ex vivo* models, such as patient-derived organoids that aim to better emulate the patient-specific TME, have limitations: they usually require significant laboratory manipulation and are still typically missing critical systemic physiological components (e.g., metabolism, circulation, exact cell population ratios) (Verduin et al., 2021).

The low likelihood of translating preclinical models into clinical benefit for patients is well known and has persisted over time. One approach that may prove to be superior to *ex vivo* patient-derived models, particularly in oncology, is studying the pharmacodynamic (PD) effects of investigational drugs directly in human patients, and importantly, in a manner that does not induce systemic toxicities. An opportunity to explore and compare PD responses in humans of one or multiple novel assets or combinations would provide the drug developer an opportunity to rule-in and rule-out drug candidates, develop stratification strategies, and hone indications early in the pipeline. Historically, there were technical, workflow, and regulatory challenges that constrained this approach. A scientifically precise, informative, and effective translational biomarker trial conducted in human patients requires 1) safe dosing of an investigational drug with minimal to low potential harms; 2) collection of high-quality, analyzable biospecimens; 3) precise confirmation of drug exposure at target sites; 4) robust and informative analytical methods; and 5) a regulatory pathway that enables this workflow. The CIVO platform was developed to specifically address these challenges (Klinghoffer et al., 2015). Herein, we provide a comprehensive review of CIVO platform use to date in human patients, a depiction of the robust analysis methods that can be coupled with this platform, and a thorough description of the operational and regulatory mechanisms used to deploy this approach.

## Materials

**FIGURE 1:**
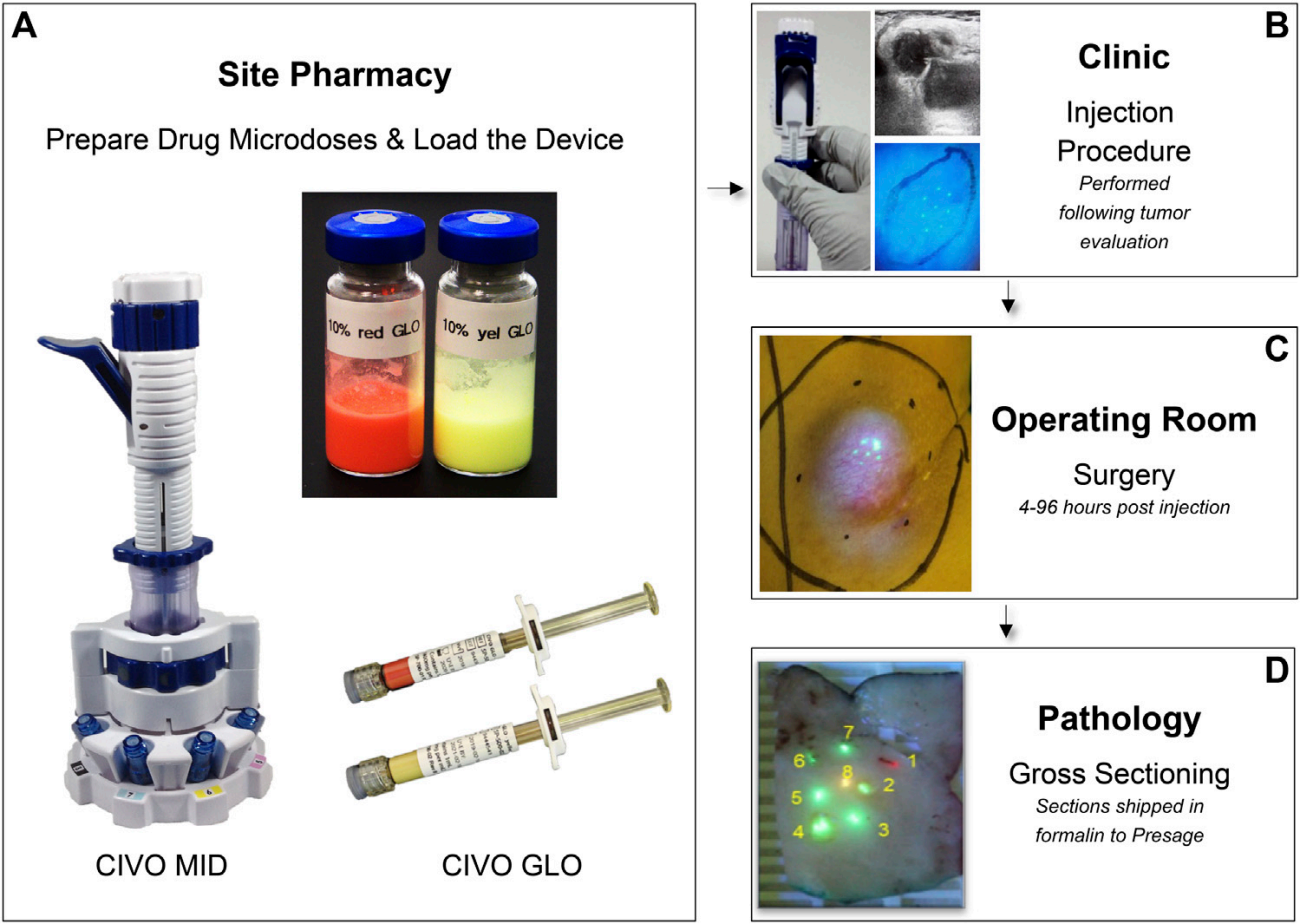
CIVO Phase 0 Clinical Workflow **(A)**. Drugs are mixed with CIVO GLO (Yellow or Red) under aseptic conditions in the site pharmacy, per study-specific instructions in vials and loaded into the MID transfer vessel using luer-lock syringes. **(B)**. Once loaded, the injector is transferred from the pharmacist to the investigator performing the injection. Immediately prior to an injection, ultrasonography is used to record the dimensions of the mass, evaluate the tumor’s internal architecture, identify the optimal injection placement, and customize the MID to the patient’s tumor (adjusting the depth of needle insertion and length of deposited column). During an injection, needles are carefully inserted into the tumor, and then, upon lever actuation, are retracted slowly within the tumor tissue to simultaneously deposit distinct and trackable drug columns containing minute volumes (up to 8.3 µL) of each drug, drug combination, or control. This is performed as an outpatient procedure. **(C)**. Following injection, 4–96 h later, the patient returns to the site for the scheduled surgical resection of the injected tumor, per the patient’s standard of care plan. **(D)**. The excised tumor is then transferred to pathology, where the injected portion of the tumor sample is identified, using custom blue light and yellow filter lens, then cut out, sectioned transverse to the injection columns in ∼4 mm sections and placed into 10% buffered formalin containing 0.92 mg/mL sodium orthovanadate, 1.5 mg/mL sodium glycerophosphate, 1 mg/mL sodium fluoride, and 2.2 mg/mL sodium pyrophosphate and shipped at room temperature.

The CIVO platform consists of a single-use, handheld microdose injector (MID), fluorescent red tracking microspheres (CIVO GLO Red) and fluorescent yellow microspheres (CIVO GLO Yellow) (Figure 1). CIVO GLO microspheres are inert polystyrene beads that are, on average, ∼4.5 microns in diameter. The MID consists of two parts: A transfer vessel into which microdose quantities of up to 8 drugs, drug combinations, or control agents are loaded following compounding with CIVO GLO, and the detachable injector component. The injector, which is configurable with three, five, or eight 25-gauge needles, can be adjusted to 1) control the needles’ reach within the tumor, and 2) set the length of the column of drug that is deposited into the tumor. The MID is designed to access tumors or lesions up to 3 cm in depth and deposit a columnar array of drugs up to 10 mm in length. Based on drug diffusion from the injected columns of approximately 1500 microns (1.5 mm) in radius (Klinghoffer et al., 2015), each column of drug represents approximately a 3 mm × 10 mm column of drug-exposed tissue (71 mm^3^). Each CIVO injection results in up to 8 drug columns, thus up to 568 mm^3^ of drug-exposed tissue available for downstream analyses as described in the Methods. Additional materials provided to clinical sites for CIVO platform use in a Phase 0 trial are listed in Supplementary Figure S1.

## Methods

Traditional early drug development involves testing large numbers of molecular derivatives, first in *in vitro* testing models, and subsequently in *in vivo* animal models, in order to select viable candidate drugs, based on binding affinities, enzymatic activities, cellular toxicity, *in vivo* efficacy, pharmacokinetics, and safety profiles. This includes additional assessments in mice, rats, dogs, and potentially non-human primates. Thereafter, an investigational new drug (IND) application (for development planned in the United States) is submitted for each viable candidate before studies can be conducted in human patients. In this traditional approach it is not until Phase 1 (at best), and sometimes Phase 2 studies, that any information on the PD effects of the drug in human tumors can be obtained.

### The phase 0 regulatory framework and microdose calculations

Recognizing the need for early evaluation of novel compounds directly in patients, the U.S. Food and Drug Administration (FDA) introduced Guidance in 2006 that provides a regulatory framework for exploratory IND (eIND) clinical trials. This Guidance forms the basis of a so-called *Phase 0* study (FDA, 2006). As defined in the Guidance, exploratory IND studies have no therapeutic or diagnostic intent, involve very limited human drug exposure and, by design, present limited risk to human patients. This regulatory framework may enable confirmation of a drug’s mechanism of action, provide pharmacokinetic (PK) information or biodistribution properties, and help prioritize candidates based on their PD or PK properties, early in the development process. While eIND studies may include single- or multiple-dose studies, dose escalations, and radiolabeled candidates, they typically evaluate subtherapeutic or limited dosing schedules.

The study design used in CIVO platform trials is to administer microdose quantities of multiple drugs directly into a patient’s tumor (either primary site or metastatic lymph node). Per the 2006 Guidance, a microdose is defined as “less than 1/100th of the dose of a test substance calculated (based on animal data) to yield a pharmacologic effect of the test substance with a maximum dose of ≤100 μg (for imaging agents, the latter criterion applies). Due to differences in molecular weights as compared to synthetic drugs, the maximum dose for protein products is ≤30 nmol” (FDA, 2006). In cases where existing data on an asset in development is available (e.g., the Recommended Phase 2 Dose (RP2D)), the calculation of the maximum CIVO microdose is straightforward. In cases where it is not yet known, the Guidance allows for microdose calculation based on preclinical efficacy and safety data.

In the Phase 0 clinical trial setting, the CIVO platform is designated as a *research tool* by the U.S. FDA. All investigational agents injected via CIVO must have reciprocal cross-reference authorization by both parties- the pharmaceutical manufacturer and Presage- prior to commencement of the planned clinical trial.

### Microdose and CIVO device compatibility

Evaluation of compatibility between the drug product(s), the microdose solution preparation, the CIVO GLO microspheres, and drug contacting surfaces of the CIVO device is included in the U.S. FDA regulatory submission. Compatibility studies are performed at room temperature mimicking the typical conditions within the compounding pharmacy and patient clinic where the microdoses are prepared and loaded into the CIVO injection device. To date, assessment of study drug product compatibility with the CIVO device has been evaluated for all microdose formulations using inspection for visible particulates per USP <790> and high-performance liquid chromatography (HPLC) methods to determine percent recovery, impurities, and/or high molecular weight (HMW) and low molecular weight (LMW) species before and after 4-h incubation with CIVO.

### Patient enrollment

Patients were enrolled in IRB-approved (feasibility or Phase 0) multi-center trials. All subjects provided informed consent and were screened for eligibility. The studies were conducted in accordance with the ethical guidelines outlined in the Declaration of Helsinki and the International Council on Harmonization guidelines on Good Clinical Practice. Inclusion and exclusion criteria varied by study, but key inclusion criteria included male and female adults (≥18 years), informed consent, and a pathologic diagnosis of the intended target tumor (e.g., head and neck cancer (HNC), head and neck squamous cell carcinoma (HNSCC), lymphoma (LSA), soft tissue sarcoma (STS)) with a surface-accessible tumor amenable to CIVO injection that is planned for surgical resection.

Eligible patients underwent microdose injection with the CIVO device on an outpatient basis.

### CIVO loading

Under aseptic conditions in the site pharmacy, drugs are mixed with CIVO GLO (Yellow or Red) in accordance with study-and patient-specific instructions. The drugs are loaded from a vial into the MID transfer vessel via luer-lock syringes. To orient the injection columns and ascribe a given site to exposure of a given agent, at least one injection site includes CIVO GLO Red (Figure 1).

### CIVO injection

Available pre-operative imaging is reviewed to confirm tumor tissue morphology, dimensions, and characteristics to identify the optimal injection site and injection angle. Immediately prior to an injection, ultrasonography can be used to record the dimensions of the target mass, evaluate the tumor’s internal architecture, and adjust the settings of the MID (Figure 1). Topical or subcutaneous pain mitigation measures may be applied prior to the injection. During an injection, needles are simultaneously inserted into the tumor, and then, upon lever actuation, are retracted slowly within the tumor tissue to simultaneously deposit distinct and trackable drug columns containing minute volumes (up to 8.3 µL) of each drug, drug combination, or control. This is performed as an outpatient procedure.

### Sample collection

Following injection, 4–96 h later, the injected tumor is resected per a patient’s standard of care plan (Figure 1). Due to the presence of CIVO GLO, the injected portion of the tumor sample is identified in the pathology suite using a blue light and yellow filter lens. The injected tissue is then sectioned transverse to the injection columns in −4 mm sections and placed into 10% buffered formalin containing 0.92 mg/mL sodium orthovanadate, 1.5 mg/mL sodium glycerophosphate, 1 mg/mL sodium fluoride, and 2.2 mg/mL sodium pyrophosphate and shipped at room temperature. Samples remain in formalin for >48 h before being formalin fixed and paraffin embedded (FFPE) and further processed for downstream histopathologic and spatial profiling analyses.

### H&E, ISH, IHC biomarker staining

Antibodies are commercially sourced and optimized for use in immunohistochemistry assays performed on CIVO-injected tissues. During immunohistochemistry (IHC) assay optimization, following antigen retrieval, multiple concentrations of the candidate primary antibody are tested on appropriately selected antigen-positive control tissues. Positive control tissues are selected based on literature search and genome/protein databases (e.g., GeneCards, NCBI, Human Protein Atlas, UniProt). Non-specific antibody binding and artefact presence are assessed via three methods: 1) parallel staining of species matched isotype antibody controls on the positive control tissues; 2) antibody staining of antigen-negative tissue controls; and 3) secondary detection antibody without primary antibody on antigen-positive and antigen-negative control tissues. IHC optimization staining is reviewed to verify the staining patterns and cellular localization of the antibody signal are specific and consistent with the literature. Following IHC assay establishment, subsequent experimental staining batches are executed and include positive control tissues and the appropriate species-matched isotype control staining. Controls for each experimental staining batch are similarly reviewed to ensure antibody performance and data reproducibility over time.

**FIGURE 2:**
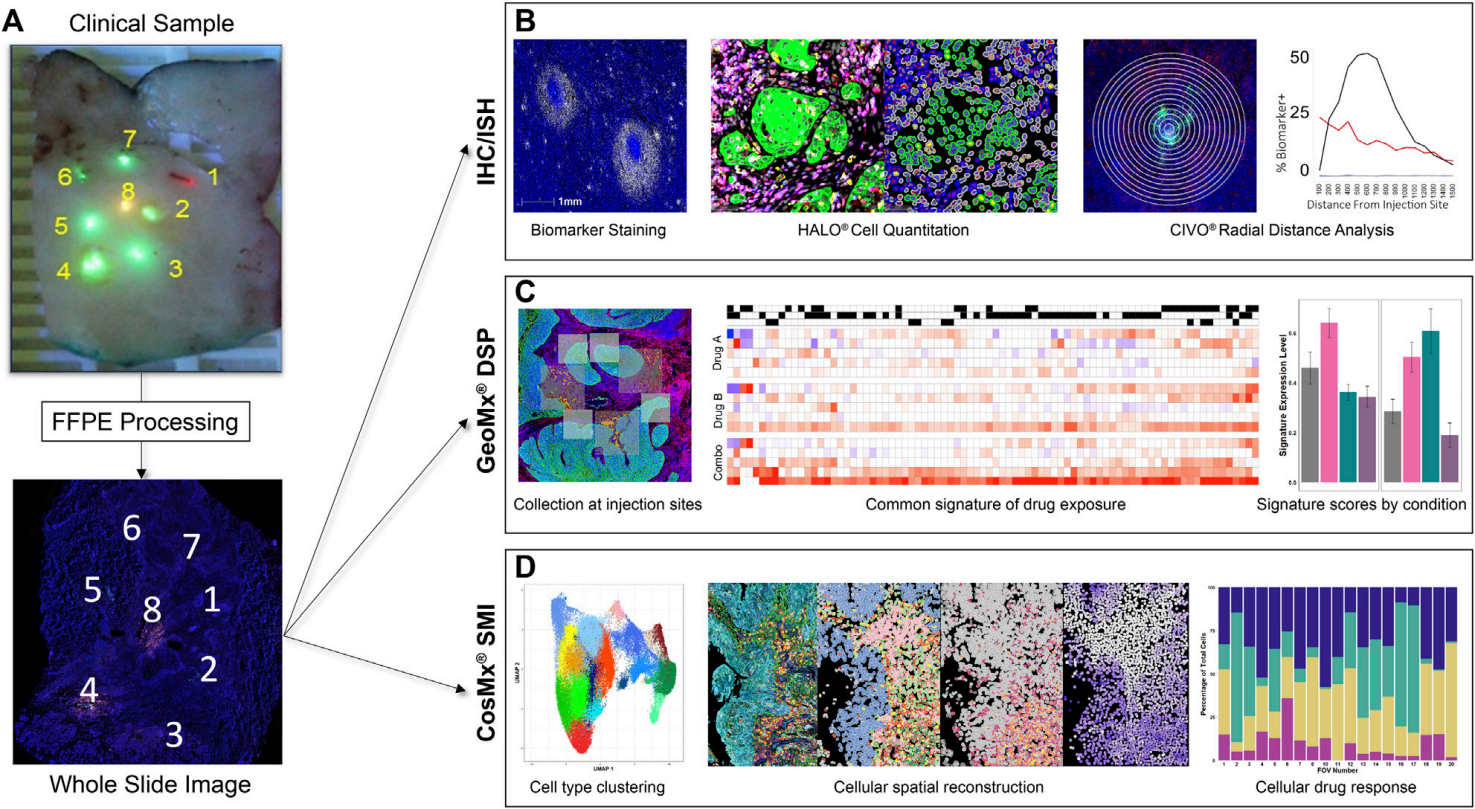
CIVO Phase 0 Analysis Workflow **(A)**. Formalin-fixed tissue sections are received by Presage, processed, embedded, and cut at 4 μm onto glass slides. **(B)**. For IHC/ISH staining, slides are stained using fluorescent detection of antibody/probe and scanned on a whole-slide scanner. Cell segmentation and biomarker analysis is performed using HALO^®^ (Indica Labs) to attain cell-level data. This is then measured over radial distance from the injection site to visualize the changes in drug effect across the gradient of drug exposure from the injection site. **(C)**. For GeoMx^®^ DSP, slides are stained for morphology markers and a GeoMx probe mix of barcoded RNA probes, then probes are photocleaved and collected from areas of interest (AOIs) around each injection site. Probe counts are quantified using next-generation sequencing and matched to their corresponding genes, which are compared across treatments to determine differentially expressed genes between injection sites. Common signatures of drug exposure are generated across patients in addition to patient specific responses. These signatures, as well as publicly available signatures, can then be used to score the injection sites by patient and drug. **(D)**. For CosMx^®^ SMI, slides are prepped with CosMx Universal Cell Characterization Panel RNA probe set, then probes are counted at a single cell level within predefined fields of view (FOVs) at each injection site. Cells are defined and clustered based on differential RNA expression. This data can then be used to spatially reconstruct the tumor areas, allowing comparisons of parallel IHC images to cell type, drug response and drug exposure. Cell type or expression data can also be used to compare FOV composition or signature scores to drug exposure quantitatively.

Resected tissues are analyzed at multiple levels along the injected columns of drug using hematoxylin and eosin (H&E), IHC, *in situ* hybridization (ISH) assays, and spatial biology analysis platforms, to track anti-neoplastic and immune-modulating activities exhibited in the TME at precise sites of drug exposure (Figure 2). FFPE CIVO-injected tumors are sectioned into 4-micron slices and placed onto slides that are then baked for 1 h at 60°C, deparaffinized in Clear-Rite 3, and rehydrated with graded alcohols. For fluorescent IHC, sections undergo antigen retrieval for 20 min at 100°C and 1-h block in 5% normal goat serum followed by primary antibody incubation overnight (Supplementary Figure S2). Next, the sections are incubated in secondary antibody conjugated to Alexafluor fluorophores, or horseradish peroxidase secondary antibody for detection using Opal fluorophores. Next, slides are counterstained with DAPI and cover-slipped with mounting medium. ISH is completed using the RNAscope multiplex fluorescent reagent kit v2 (Advanced Cell Diagnostics). Whole slide images are then acquired using a digital, automated, high-resolution scanner. To date, CIVO clinical trial samples have been stained and evaluated with 72 IHC and 43 ISH assays (Supplementary Figure S2).

### Pathologist review

Additionally, H&E stains of representative sections from each tumor are submitted for review by board-certified anatomic pathologists who are blinded to the contents at each injection site. This blinded review is used to help confirm or clarify observations from the above-described analytical methods.

### GeoMx digital spatial profiling

Transcriptomic and single-cell analyses using digital spatial profiling techniques have also been applied to FFPE tissues resulting in thousands of RNA and protein molecules being probed to identify clusters of response (or non-response) within and between tissues, drugs, and controls (Derry et al., 2023; Gundle et al., 2023; Rajasekaran et al., 2023). GeoMx methods have been described elsewhere (Derry et al., 2023). Briefly, slides are incubated overnight with Human Cancer Transcriptome Atlas probe mix (NanoString #121400101), then stained with IHC antibodies for panCK and CD45 and Syto13 for nuclear detection. Following whole slide imaging, multiple geometric Regions of Interest (ROIs) of 400 µm^2^ are placed at each injection site. Indexing oligonucleotides are then cleaved, collected, processed and quantified via next-generation sequencing on a NovaSeq 6000 Illumina sequencer as previously described (Zollinger et al., 2020).

### CosMx spatial molecular imaging

CosMx cyclic RNA readout is performed on tissue sections as previously described (He et al., 2022). Briefly, tissue sections are dewaxed, rehydrated, then undergo target retrieval, protease digestion, and post-fixation. Hybridization chambers are applied to the slides and the probe set incubated on the tissue overnight. Un-bound probe is removed through stringency washes. Nanostring flow cells are applied to the slides and loaded onto the SMI instrument. Field of views are selected that correspond to the GeoMx ROI on parallel tissue sections, and cyclic RNA readouts are acquired. Following RNA readouts, the tissue sections are stained using a four-antibody cocktail stain of morphology markers (B2M, CD45, CD3, and PanCK) and imaged on the SMI instrument.

### Spatial biology data analysis

All analyses for GeoMx DSP and CosMx SMI are performed using R in RStudio. In general, the analytical goal for both technologies is to distinguish transcriptional changes induced by drug exposure (PD effects) by comparing ROIs adjacent to drug injection sites to those adjacent to vehicle or background (non-injected) sites. For GeoMx DSP, raw probe level counts from the NanoString DSP machine are subjected to QC and normalization as previously described (Derry et al., 2023). Following QC, differentially expressed genes between drug and vehicle sites are identified using a standard *limma* pipeline on TMM-normalized probe-level data (Ritchie et al., 2015). Use of the *limma* framework allows linear models to be fit to account for variables such as patient, tumor section level, and ROI replicate, thereby extracting variance associated with drug treatment. In some cases, where tumor heterogeneity is very high, it is necessary to account for the cellular differences across ROIs. Then cell type percentages are estimated using a spatial deconvolution function (Danaher et al., 2022). Clustering is performed using ComplexHeatMap (Gu et al., 2016) and pathway signatures derived using the gene set variation analysis (GSVA) algorithm (Hänzelmann et al., 2013).

NanoString CosMx SMI data, which consist of a sparse matrix of gene expression counts, a data frame of centroids (cell_ids and x, y coordinates), and a data frame of molecule pixel coordinates (gene_ids and x, y coordinates), are processed as previously described (Derry et al., 2023). The LoadNanoString function is used to load data in R, including coordinates for the cell boundaries. The data is then quality controlled, gene counts are normalized, and Uniform Manifold Approximation and Projection (UMAP) is performed according to the vignette provided by the authors of Seurat (https://satijalab.org/seurat/articles/spatial_vignette_2). Gene markers to distinguish UMAP clusters are identified using the *FindAllMarkers* function and cell identities manually assigned to the clusters by reference to established marker sets. GSVAfor the CosMx data is performed using the *enrichIt* function (Borcherding et al., 2021).

## Results

### Feasibility clinical trials with approved agents

After extensive validation in preclinical models, use of the CIVO platform and workflow was assessed clinically in a 4-patient first-in-human LSA study initiated in 2012 (NCT01831505) (Klinghoffer et al., 2015). A second study (NCT03056599) enrolled 23 patients presenting with STS (Gundle et al., 2020). In both studies, approved agents (Table 1) were administered via CIVO MID injection into tumor tissue (lymph node (NCT01831505) or primary mass (NCT03056599)). The objectives of these trials were to establish that 1) the clinical workflow could be reasonably integrated into patient care, 2) use of the platform was safe, 3) injected tissue could be successfully recovered, 4) discrete injection columns could be resolved in tumor samples, and 5) PD analysis could be performed. All objectives were achieved in these two feasibility trials (demographics and safety data are included in the summary provided in Tables 2, 3) and use of the platform was deployed to assess PD effects of novel agents on the tumor microenvironment.

**TABLE 1:** Characteristics of drugs administered across all trials via CIVO injection (asterisks (*) identify drugs included in combinations, hashtags (#) identify drugs for which full CIVO-drug compatibility evaluation was performed, with data reviewed by FDA in an eIND submission). STS = soft tissue sarcoma; LSA = lymphosarcoma; HNC = head and neck cancer; HNSCC = head and neck squamous cell carcinoma.

Study NCT#	Drug	Category	Development status	Cancer type	Subjects administered
**NCT01831505/NCT03056599**	Doxorubicin*	Anthracycline, chemotherapeutic, small molecule	Approved	LSA, STS	4 LSA 23 STS
**NCT01831505**	Vincristine*	Vinca alkaloid, chemotherapeutic, small molecule	Approved	LSA	4 LSA
**NCT01831505**	Prednisolone*	Corticosteroid, small molecule	Approved	LSA	2 LSA
**NCT03056599**	Trabectedin	Alkylating agent, chemotherapeutic, small molecule	Approved	STS	19
**NCT03056599**	Gemcitabine	Anti-metabolite, chemotherapeutic, small molecule	Approved	STS	14
**NCT03056599/** **NCT04272333**	Nivolumab*^#^	Human IgG4 anti-PD-1, monoclonal antibody	Approved	STS, HNSCC	10 STS 1 HNSCC
**NCT03056599**	Atezolizumab	Humanized IgG1 anti-PD-L1, monoclonal antibody	Approved	STS	2
**NCT03056599**	Durvalumab	Humanized IgG1 kappa anti-PD-L1, monoclonal antibody	Approved	STS	1
**NCT03056599**	Olaratumab*	Humanized IgG1 anti-PDGFRα, monoclonal antibody	Approved	STS	9
**NCT03056599**	Aldesleukin*	Recombinant human interleukin-2, cytokine	Approved	STS	12
**NCT04272333**	Motolimod*^#^	Toll-like receptor 8 agonist, small molecule	Investigational (Ph II)	HNSCC	1
**NCT04541108**	Pembrolizumab*^#^	Humanized IgG4 anti-PD-1, monoclonal antibody	Approved	STS, HNSCC	6 STS 7 HNSCC
**NCT04541108**	MK-0482*^#^	Humanized IgG4, anti-ILT3, monoclonal antibody	Investigational (Ph I/II)	STS, HNSCC	6 STS 7 HNSCC
**NCT04541108**	MK-4830*^#^	Human IgG4, anti-ILT4, monoclonal antibody	Investigational (Ph I/II)	STS, HNSCC	6 STS 7 HNSCC
**NCT04065555**	Avelumab*^#^	Human IgG1 anti-PD-L1, monoclonal antibody	Approved	HNC	10
**NCT04065555**	Cetuximab*^#^	Chimeric IgG1 anti-EGFR, monoclonal antibody	Approved	HNC	9
**NCT04065555**	Subasumstat*^#^	SUMOylation inhibitor, small molecule	Investigational (Ph I/II)	HNC	12
**NCT06062602**	5-Fluorouracil*^#^	Anti-metabolite, chemotherapeutic, small molecule	Approved	HNSCC	11
**NCT06062602**	Carboplatin*^#^	Platinum-based chemotherapeutic, small molecule	Approved	HNSCC	15
**NCT06062602**	Paclitaxel*^#^	Taxane, chemotherapeutic, small molecule	Approved	HNSCC	15
**NCT06062602**	TAK-676*^#^	STING agonist, small molecule	Investigational (Ph I)	HNSCC	15

**TABLE 2:** Patient demographics by CIVO study.

Study	Total enrolled	Age mean	Age min	Age max	Gender #	Ethnicity # (%)	Race # (%)
**NCT01831505**	4	49	25	65	M: 50% (2/4)	Hispanic or Latino: 25% (1/4)	White: 75% (3/4)
					F: 50% (2/4)	Not Hispanic or Latino: 75% (3/4)	Black or African: 25% (1/4)
**NCT03056599**	23	55	28	93	M: 61% (14/23)	Hispanic or Latino: 9% (2/23)	White: 74% (17/23)
					F: 39% (9/23)	Not Hispanic or Latino: 82% (19/23)	Black or African: 9% (2/23)
						Unknown/Not Reported: 9% (2/23)	Not Reported: 17% (4/23)
**NCT04272333**	1	61	61	61	M: 100% (1/1)	Hispanic or Latino: 100% (1/1)	White: 100% (1/1)
					F: 0% (0/1)		
**NCT04065555**	12	63.5	42	87	M: 83% (10/12)	Not Hispanic or Latino: 92% (11/12)	White: 100% (12/12)
					F: 17% (2/12)	Unknown/Not Reported: 8% (1/12)	
**NCT04541108**	13	68.3	38	87	M: 85% (11/13)	Not Hispanic or Latino: 77% (10/13)	White: 77% (10/13)
					F: 15% (2/13)	Unknown/Not Reported: 23% (3/13)	Native Hawaiian or Pacific Islander: 8% (1/13)
							Not Reported: 15% (2/13)
**NCT06062602**	15	64.1	48	72	M: 93% (14/15)	Hispanic or Latino: 20% (3/15)	White: 73% (11/15)
					F: 7% (1/15)	Not Hispanic or Latino: 60% (9/15)	Not Reported: 27% (4/15)
						Unknown/Not Reported: 20% (3/15)	

**TABLE 3:** CIVO-Related adverse events reported across all trials (As of December 2023).

Description	Grade	Frequency	Relatedness	Outcome
**Injection Site Pain**	1	10/68 (14.7%)	Possibly, Probably, or Definitely Related to Injection Procedure	Resolved without sequelae
**Injection Site Bleeding**	1	1/68 (1.5%)	Definitely Related to Injection Procedure	Resolved without sequelae
**Injection Site Bruising**	1	1/68 (1.5%)	Probably Related to Injection Procedure	Resolved without sequelae
**Dysgeusia**	1	1/68 (1.5%)	Possibly Related to Microdose Injection Contents	Resolved without sequelae
**Nausea/Vomiting**	1	1/68 (1.5%)	Possibly Related to Microdose Injection Contents	Resolved without sequelae
**Cellulitis**	2	1/68 (1.5%)	Possibly related to Microdose Injection	Resolving at study end

### Phase 0 clinical trials with novel agents

As of December 2023, the CIVO Platform has been used by 3 pharmaceutical partners to evaluate 5 investigational agents, 7 approved drugs, and combinations thereof (NCT04272333, NCT04065555, NCT06062602, NCT04541108) in four Phase 0 clinical trials, enrolling a total of 41 subjects. The first two of these trials were designed as stand-alone studies, each with their own protocol, eIND, and site network. However, given that the workflow for any CIVO-platform trial is nearly identical (i.e., workflow and study procedures are the same, the study drugs differ), an umbrella-concept trial (PBI-MST-01) was initiated. As of December 2023, 10 sites located in the United States have IRB-approval on this umbrella trial, and two substudy cohorts have been completed.

### Outcomes from all CIVO trials: demographics, compatible drug classes, and safety

In total, the CIVO platform has been used in 6 clinical trials (2 feasibility studies and 4 Phase 0 studies), enrolling 68 subjects across three indications: LSA, STS, and HNC. Table 2 provides a summary of patient demographics and tumor types for patients enrolled to date.

Owing to the CIVO MID’s multiplex design (up to 8 needles per injection), a total of 21 individual drugs, and 13 drug combinations have been evaluated (Table 1, with drugs that have been included in combinations identified with an asterisk). Note that this includes chemotherapeutics, small molecule inhibitors/agonists, and monoclonal antibodies administered singly and in combination (e.g., monoclonal antibody + chemotherapeutic).

**FIGURE 3:**
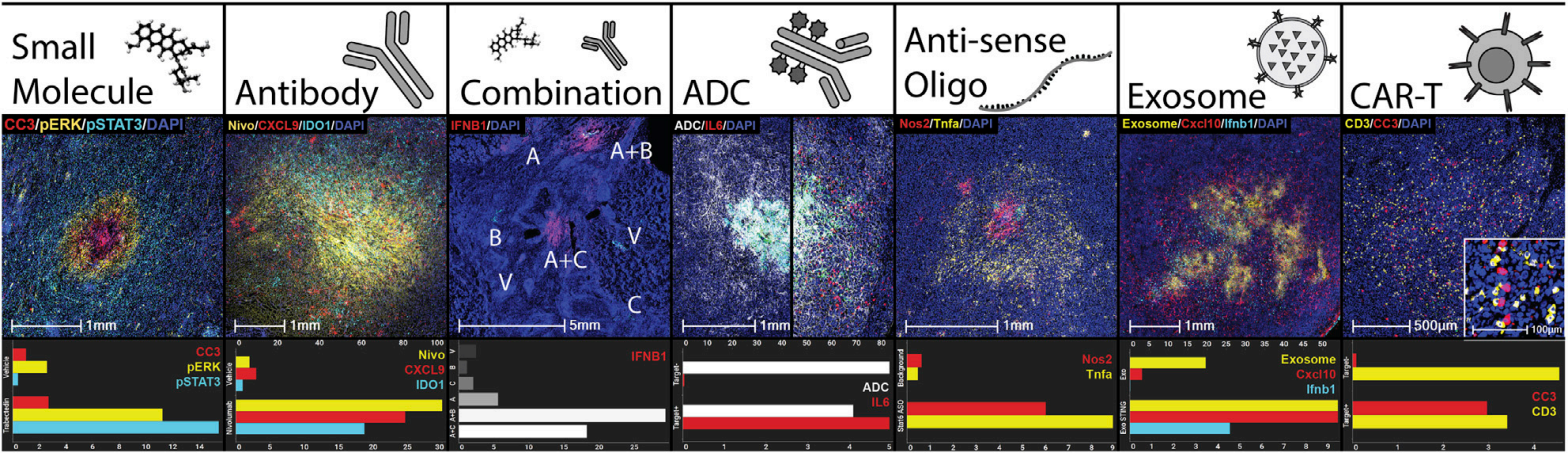
CIVO Compatibility with Classes of Drugs. Demonstration of delivery and efficacy of drug classes using the CIVO platform. Each column is an example of a drug class delivered clinically or preclinically, as well as sample quantitation using HALO^®^ of the pictured injection site compared to controls. Quantitation is % positivity unless otherwise stated. Small Molecule: Trabectedin injection site in a clinical soft tissue sarcoma patient, stained for cleaved caspase 3 (IHC, CC3, red), phospho-ERK (IHC, pERK, yellow), phospho-STAT3 (IHC, pSTAT3, cyan) and nuclei (DAPI, blue). Antibody: Nivolumab injection site in a clinical soft tissue sarcoma patient, stained for nivolumab (IHC, Nivo, yellow), CXCL9 (ISH, red), IDO1 (ISH, cyan) and nuclei (DAPI, blue). Top axis is Nivolumab % positive. Combination: Vehicle (V), subasumstat (A), cetuximab (B), avelumab (C), and combination (A + B, A + C) injection sites in a clinical head and neck squamous cell carcinoma patient, stained for IFNB1 (ISH, red). Antibody-Drug Conjugate (ADC): Injection sites containing a tumor-targeted, myeloid-stimulating linker-payload drug in preclinical humanized xenograft models, one negative (left) and one positive (right) for the antibody target. Stained for ADC distribution (IHC, ADC, white) and IL6 (ISH, red). Top axis is ADC % positive. Anti-sense Oligo (ASO): Stat6 ASO injection site in preclinical murine melanoma model stained for Nos2 (ISH, red), Tnfa (ISH, yellow), and nuclei (DAPI, blue). Top axis is % Nos2 positive. Control is a background region. Exosome: Injection site for an exosome containing STING agonist in preclinical murine lymphoma model stained for an exosome surface protein (IHC, Exosome, yellow), Cxcl10 (ISH, red), Ifnb1 (ISH, cyan), and nuclei (DAPI, blue). Top axis is % Exosome positive. Control is empty exosome. CAR-T: Human CAR-T injection site, in a preclinical xenograft model positive for CAR-T target, stained for CD3 (IHC, yellow), cleaved caspase 3 (IHC, CC3, red), and nuclei (DAPI, blue). CC3 is represented as percent positive as a fold change over the vehicle injection site. For the control site, the same CAR-T cells were injected in a preclinical xenograft model negative for CAR-T target.

To maximize the potential for testing broad classes of injectable drugs via multiplex microdosing, the CIVO injector and CIVO GLO microspheres were designed and manufactured using materials that are non-reactive and have low drug adsorption properties. To date, all classes of drugs tested (preclinically and/or in Phase 0) have been found to be compatible with the CIVO device and CIVO microinjection procedure. This includes traditional cytotoxics/chemotherapeutics, RNA drugs, nanoparticles, antibodies, antibody-drug conjugates, bispecifics, cell-based therapies. Table 1 and Figure 3 illustrate the compatibility of the CIVO platform with many of these drug classes.

Importantly, the CIVO platform has been demonstrated as safe across 68 patients and 3 indications tested to date. Injection-related adverse events were limited to low frequency, mild (14/68 patients; 20.6%) to moderate (1/68 patients; 1.5%) events which resolved without sequelae (Table 3). While injection site pain was reported in 14.7% (10/68) of patients, these specific events occurred during the early clinical feasibility studies. Pain has since been mitigated through improved local anesthesia protocols, including application of eutectic mixture of local anesthetics (EMLA) cream and injection of subcutaneous lidocaine, and no pain-related adverse events have been reported since. To date, there have been no Serious Adverse Events assessed as related to the CIVO injection procedure or the injection contents. Overall, the risk profile has been and continues to be assessed as low.

### Efficacy outcomes summary: studies with investigational agents

Depending on the stage of development of the drug, CIVO Phase 0 studies can be optimized to provide different types of data. Although data is available from all 4 Phase 0 studies, only previously published data could be referenced below.

For drugs without existing INDs, for which a CIVO Phase 0 study represents a first-in-human trial, in-depth analyses of a novel agent’s impact on the TME enable confirmation or rejection of the drug’s presumptive mechanism of action and/or PD impact on the human TME. An upcoming study is being initiated within this framework. This study, which includes investigation of a pre-GMP agent (PBA-0405), received an FDA “Study May Proceed” letter in December 2023. We now have additional clarity as to the reduced US FDA Chemistry, Manufacturing, and Controls (CMC) and preclinical safety data requirements for pre-IND drug product to be evaluated in Phase 0 studies.

CIVO Phase 0 studies can also uncover critical information about the performance of drugs with existing INDs already in Phase 1/2 trials, and possibly ahead of Phase 1 results. For example, CIVO Phase 0 (NCT04065555) data delivered first-of-its-kind evidence of the immune effects of Subasumstat in HNC patient tumors (Derry et al., 2023). Additionally, in the most recent CIVO Phase 0 study (NCT04541108) and in contrast to preclinical data, CIVO data in human HNSCC patients demonstrated that localized TME exposure to pembrolizumab in combination with either of two myeloid derived suppressor cell-targeting agents, anti-immunoglobulin-like transcript 3 (ILT3) mAb MK-0482 or anti-ILT4 mAb MK-4830, did not enhance the immune response induced by pembrolizumab alone in HNSCC or STS (Gundle et al., 2023).

Moreover, while the timeframe from injection to resection may seem limited (4–96 h), CIVO data in human patients can successfully capture both apoptotic and immune modulation activity over this time period. This was demonstrated in NCT04065555, a study designed to evaluate the investigational drug subasumstat in HNC over several timepoints (Derry et al., 2023). When multiple drug candidates are evaluated in parallel following a CIVO injection, the data can also facilitate pipeline prioritization based on the PD data obtained. Finally, CIVO studies can serve as indication-finding exploratory studies or biomarker-identifying studies to help decipher which cancer types/subtypes or patient subgroups may be more or less likely to respond to therapies evaluated in future Phase 2 trials and beyond.

## Discussion and future directions

The CIVO platform was designed to improve cancer drug development by replacing efficacy studies performed in non-human animal models with early and safe PD studies performed directly in the only relevant context: the human cancer patient. Thus far, use of the platform has provided an entirely novel mechanism to inform and de-risk drug development pipeline programs, while minimizing risk to patients. By offering drug developers this alternative, use of the platform is directly aligned with the FDA Modernization Act 2.0 (Rand et al., 2022), which includes a provision (The Reducing Animal Testing Act) that eliminates a federal mandate for animal testing for new drugs. This shift in focus from non-human animals to the human patient is overdue and should result in significantly more cost-efficient, time-saving, and successful drug development strategies. This approach could be critical not only to lowering the overall cost of healthcare, but also to enabling continued periods of strong, optimally targeted innovation, even in the face of restricted funding (start-up environments, economic recessions, etc.).

CIVO Phase 0 studies present multiple advantages when compared to preclinical efficacy modeling, as well as distinct opportunities relative to traditional Phase 1 trials (Table 4). Paramount among these distinctions is the fundamental and ongoing lack of translatability of preclinical animal studies. The complexity of the human TME, within the context of an intact human immune system, cannot be accurately modeled in a non-human species. The CIVO approach cannot replace traditional Phase 1 trials that collect critical in-human PK, biodistribution, dosing, and safety information. However, an antecedent Phase 0 study can be used to demonstrate whether a candidate asset elicits the intended on-target responses and/or downstream effects, based on its anticipated mechanism of action. This evaluation can be used to stage-gate drugs to proceed through the pipeline - or not -, sparing the considerable cost, as well as patient risks, associated with candidate drugs that do not work.

**TABLE 4:** Comparison of early-phase efficacy testing.

CIVO phase 0 trial	Traditional preclinical efficacy modeling	Cancer organoids and other more recently developed *Ex Vivo* models	Traditional phase 1 trial
**Collection of in-depth PD and TME data; could enable biomarker selection and validation**	Collection of efficacy data, but does not enable full recapitulation of the human TME; biomarker selection limited; validation not possible until in-human studies	Collection of efficacy data possible, but *ex vivo* *;* incomplete and inexact recapitulation of the human TME	Some Ph1 trial designs enable limited preliminary biomarker evidence of PD effects and early identification of patient population
**Minimal risk to the human patient**	No risk to the human patient	No risk to the human patient	Considerable risk to human patient
**Early PD information on investigational agents enabling asset prioritization and indication selection**	Nonclinical asset prioritization and indication selection possible but at risk due to lack of or poor translatability of many preclinical models	Non-clinical asset prioritization and indication selection possible but at risk due to inexact translatability	Prioritization of assets must happen prior to Phase I initiation; selection of indication typically done through nonclinical assays
**Sampling potentially thousands of TME responses via columnar delivery of drug(s)**	No human TME response data collected	Collection of efficacy data possible, but incomplete and inexact recapitulation of the human TME	May collect limited preliminary PD data
**Drug exposure in tumor confirmed by CIVO GLO tracking microspheres**	No true human tumor drug exposure evaluated	Drug exposure can be confirmed	Unless special assays used, no confirmation of tumor exposure
**Compatible with all drug types (cytotoxics, RNA drugs, nanoparticles, protein therapeutics, cell therapy, etc.) except prodrugs requiring extra-tumoral metabolism for activation**	Compatibility depends on interspecies drug target consistency	Compatible with all drugs, except prodrugs requiring extra-tumoral metabolism for activation	Compatible with all drug types
**Independent of PK or biodistribution concerns**	Does not provide PK and biodistribution data in humans	Does not provide PK and biodistribution data in humans	Provides PK +/- biodistribution data in humans
**Minimal toxicology package required; potential for non-GLP data inclusion**	No toxicology package required	No toxicology package required	Traditional GLP toxicology package required
**Pilot cGMP lot product acceptable as well as appropriately controlled pre-GMP material**	Non-GMP material acceptable for studies in nonclinical models	Non-GMP material acceptable	cGMP clinical lot required
**Multiplexing of single agents and combinations enabling comparative analysis within the same patient**	No multiplexing of single agents possible within an animal; combinations evaluated through multiple study arms	Only one drug or drug combination is typically assessed per structure, but larger scale drug screening possible	Only one drug or drug combination is typically assessed
**In-depth analysis of PD and human TME, in drug-exposed and non-exposed areas**	Accuracy of human PD or TME analysis in preclinical models questionable	Comparison between treatment groups possible, but requires multiple structures	Analysis of PD and human TME limited

Ultimately, however, the PD effects observed following human intratumoral microdosing need to be correlated with systemic outcomes. This presents both the most significant current limitation as well as the greatest opportunity of this platform. In the best use-case scenario, the platform could be applied along the entire drug development continuum to 1) rule-in and rule-out drug candidates based on intratumoral response; 2) evaluate PD differences in on- and off-target responses and develop differentiating companion biomarker profiles; 3) use responder profiles to stratify drug validation studies, selecting only those indications or patients most likely to benefit from the drug; and 4) ultimately deploy use of the platform as a companion diagnostic, once validated. The efficiencies of this alternative drug development model are economical, ethical, and practical. The cost of candidate assets is reduced as poor-performing assets exit the pipeline early and patients who are unlikely to benefit from systemic dosing of a drug—and could potentially endure significant toxicity—are not included in development trials. There is significant research ongoing in the development of new immunotherapies, targeted therapeutics, and combinatorial strategies in all three of these indications (Lulla and Heslop, 2016; Banks and D’Angelo, 2022; Ettl et al., 2022), and the CIVO platform is currently being used in both HNSCC and STS to evaluate novel immunomodulating agents and/or novel combinatorial strategies. Ultimately, this drug development model is one that has the potential to help achieve the societal goal of delivering the right drug to the right patient.

From a technological perspective, there are constraints worth noting. At present, the platform is limited to surface-accessible injections (−3 cm) and, thus far, has only been used in LSA, STS, and HNC. Through expanding collaborations, feasibility of CIVO platform use is also currently being explored in new indications, including breast cancer and metastatic melanoma. Future development initiatives include device design modifications to incorporate biopsy instrument features and dimensions, and device innovation to miniaturize the platform. The ultimate goal of these development efforts is to enable compatibility of the injector with all tumors, located anywhere within the human body (i.e., deep reach, or scope-based).

In summary, the safety and feasibility of the CIVO Phase 0 Platform have been established and the data collected to date have provided direct evidence of PD activity for several pharmaceutical partners’ pipeline programs. Moreover, leveraging spatial profiling analysis tools significantly augments the ability of the CIVO platform to differentiate response profiles between tissue samples, drugs, and drug combinations. Taken together, a Phase 0 trial using this approach presents pharmaceutical innovators with a robust alternative to a system of drug development that is known to be inefficient, imprecise, resource intensive, and one in which failures are the norm. A CIVO Phase 0 trial represents renewed possibility for the established drug development paradigm; drug developers must in turn both recognize the limitations of the *status quo* and be willing to innovate by integrating a Phase 0 study into their asset development plans.

## Data Availability

The original contributions presented in the study are included in the article/Supplementary Material, further inquiries can be directed to the corresponding author.
