# Supplementary material for: Early, precise, and safe clinical evaluation of the pharmacodynamic effects of novel agents in the intact human tumor microenvironment
Source: Front Pharmacol. 2024 Apr 12;15:1367581. doi: 10.3389/fphar.2024.1367581 (PMC11048044; doi:10.3389/fphar.2024.1367581)
Supplement: Supplementary file 1 [file Table1.docx]

Supplemental Table 1. Additional Materials Provided to Clinical Sites

| Material | Manufacturer | Specifications |
| --- | --- | --- |
| 2mL Vials | Adelphi | SCHOTT Neutral Type I Fiolax glass, Internally sterile and particle free. Inert internal Nitrogen at 800-900 mBar, 13mm Flip off seal, Lyo Rubber stopper Bromobutyl. |
| 5mL Vials | Adelphi | SCHOTT Neutral Type I Fiolax glass, Internally sterile and particle free. Inert internal Nitrogen at 800-900 mBar, 20mm Flip off seal, Lyo Rubber stopper Bromobutyl. |
| Xite Flashlight | NiteSea | LiOn Battery powered flashlight 440-460nm royal blue excitation w/ 500nm Longpass filter glasses |
| 15x Phosphatase Inhibitor (PPi) | Presage | 10% Neutral Buffered Formalin, Sodium Orthovanadate (1.3792% w/v), Beta-Glycerophosphate disodium salt hydrate (2.2958% w/v), Sodium Fluoride (1.575% w/v), Sodium Pyrophosphate decahydrate (3.3458% w/v), in 40mL sealed conical tube. |
| Guide Needle | Presage | 22-gauge Guide needle, guide needle 'O' ring, 16-gauge guide needle sheath, guide needle cap |
| Specimen Cups | Starplex | 90 mL Sterile, polypropylene w/ polyethlene closure, leakproof Specimen Container |
| Insulated Shipper | Various (Uline, Polar tech) | 6x5x6 1/2" Polysterene foam primary container, with corrugated cardboard outershipping container. |
| Butterfly Ultrasound | Butterfly IQ | Butterfly IQ+ w/ lightning connection, 9000 element CMUT, 1-10 MHz |
| Sonostar Ultrasound | Sonostar | Sonostar L6C Color Doppler Ultrasound Probe 7.5/10MHz L40 w/ 192 elements 32 Transmitting channels |
