# Supplementary material for: Early, precise, and safe clinical evaluation of the pharmacodynamic effects of novel agents in the intact human tumor microenvironment
Source: Front Pharmacol. 2024 Apr 12;15:1367581. doi: 10.3389/fphar.2024.1367581 (PMC11048044; doi:10.3389/fphar.2024.1367581)
Supplement: Supplementary file 2 [file Table2.docx]

**Supplemental Table 2. Table of IHC and ISH Markers used to evaluate human CIVO trial samples**

| IHC | Vendor | Catalog # | RRID | Target |
| --- | --- | --- | --- | --- |
| B2M | Cell Signaling Technology | 12851 | AB_2716551 | Antigen presenting cell |
| B7-H3 | Cell Signaling Technology | 14058 | AB_2750877 | Checkpoint inhibition |
| BCL-2 | Abcam | ab182858 | AB_2715467 | Apoptosis |
| CC3 | Cell Signaling Technology | 9661 | AB_2341188 | Apoptosis |
| CCR2 | Abcam | ab176390 | Unregistered | Chemokine receptor |
| CD11b | Cell Signaling Technology | 49420 | AB_2799357 | Myeloid cell |
| CD11c | Cell Signaling Technology | 45581 | AB_2799286 | Dendritic cell |
| CD137 | Abcam | ab252559 | Unregistered | Immune checkpoint |
| CD163 | Novus Biologicals | NB110-59935 | Unregistered | Macrophage localization |
| CD20 | Abcam | ab9475 | AB_307267 | B cell |
| CD206 | Abcam | ab64693 | AB_1523910 | Macrophage polarization: M2 |
| CD3 | Abcam | ab11089 | AB_2889189 | Pan T cell |
| CD31 | Cell Signaling Technology | 85873 | Unregistered | Vasculature |
| CD4 | Abcam | ab133616 | AB_2750883 | T cell: helper |
| CD40 | Abcam | ab13545 | AB_1951619 | Antigen presenting cell, B cell, dendritic cell |
| CD44 | Abcam | ab157107 | AB_2847859 | T cell: memory cell |
| CD45 | Leica Biosystems | NCL-L-LCA | AB_563579 | Pan-leukocyte |
| CD45RA | Abcam | ab231426 | Unregistered | Dendritic cell: plasmacytoid |
| CD56 | Cell Marque | 156R-9 | AB_2941091 | NK cell |
| CD68 | Dako | M081401-2 | Unregistered | Macrophage |
| CD8 | Abcam | ab17147 | AB_443686 | T cell |
| CD86 | Cell Signaling Technology | 91882 | AB_2797422 | Macrophage polarization: M1 |
| Cetuximab | GenScript | A01993 | Unregistered | Drug distribution, anti-idiotype |
| Cisplatin | Abcam | ab103261 | AB_10715243 | Drug distribution |
| CK | Millipore | MAB3412 | AB_94853 | Localizes the tumor epithelium |
| CRT | Abcam | ab2907 | AB_303402 | Pro-phagocytic marker |
| Durvalumab | GenScript | A02010-40 | Unregistered | Drug distribution, anti-idiotype |
| EGFR | Cell Signaling Technology | 4267 | AB_2895042 | Growth factor receptor |
| FAP | Novus Biologicals | NBP2-66844 | Unregistered | Fibroblasts |
| FOXP3 | Cell Signaling Technology | 98377 | AB_2747370 | Regulatory T cell |
| GammaH2AX | Novus Biologicals | NB100-2280 | Unregistered | DNA damage |
| GLUT1 | Abcam | ab15309 | AB_301844 | Hypoxia |
| Gr-1 | BioLegend | 127602 | AB_1089180 | Neutrophil |
| GrB | Abcam | ab134933 | AB_2889221 | NK cell, T cell |
| IDO | Cell Signaling Technology | 86630 | AB_2636818 | Dendritic cell, tumor cell |
| IL2 | Abcam | ab92381 | AB_10564139 | Cytokine |
| iNOS | Abcam | ab15323 | AB_301857 | Macrophage polarization |
| Ki67 | Abcam | ab16667 | AB_302459 | Proliferation |
| Mac387 | Dako | M0747 | Unregistered | Macrophage, monocyte, neutrophil |
| MCL1 | Cell Signaling Technology | 39224 | AB_2799149 | Apoptosis |
| MHC2 | Agilent | M0775 | AB_2313661 | Antigen presenting cell |
| MIL113 | Millenium | MIL-113-67-2 | Unregistered | Millenium, detects TAK-981 adduct |
| MPO | Leica | NCL-L-MYELO | Unregistered | Neutrophil marker |
| Nectin-4 | Abcam | ab192033 | Unregistered | Tumor marker |
| NF-kB-p65 | Cell Signaling | 8242 | AB_10859369 | NF-kB pathway |
| Nivolumab | GenScript | A01931 | Unregistered | Drug distribution |
| OX40 | Abcam | ab203220 | Unregistered | T cell |
| p16 | BioCare | ACI3231 | AB_2942034 | HPV marker |
| p4EBP1 | Cell Signaling Technology | 2855 | AB_560835 | mTOR pathway |
| pAkt | Cell Signaling Technology | 4060 | AB_2315049 | PI3K/Akt pathway |
| PCNA | Cell Signaling Technology | 2586 | AB_2160343 | Proliferation |
| PD-1 | Cell Signaling Technology | 86163 | AB_2728833 | Checkpoint inhibition |
| PD-L1 | Cell Signaling Technology | 13684 | AB_2687655 | Checkpoint inhibition |
| Pembrolizumab | GenScript | A01846 | Unregistered | Drug distribution, anti-idiotype |
| pERK | Cell Signaling Technology | 4370 | AB_2315112 | MAPK pathway |
| PGP9.5 | Abcam | ab15503 | AB_301912 | Neuronal marker |
| pHH3 | Cell Signaling Technology | 9701 | AB_331535 | Mitotic arrest |
| pIRF3 | Cell Signaling Technology | 29047 | AB_2773013 | STING pathway |
| pMPM2 | Millipore | 05-368 | AB_309698 | Proliferation |
| pPDGFRa | Abcam | ab5460 | AB_304906 | Growth factor receptor |
| pS6 | Cell Signaling Technology | 4858 | AB_916156 | mTOR pathway |
| pSTAT1 | Cell Signaling Technology | 9167 | AB_561284 | BTK pathway, Jak/STAT pathway |
| pSTAT3 | Cell Signaling Technology | 9145 | AB_2491009 | MAPK pathway, mTOR pathway |
| pSTAT5 | Abcam | ab32364 | AB_778105 | Growth pathway, immune pathway |
| pTBK1 | Cell Signaling Technology | 5483 | AB_10693472 | STING pathway |
| S100A9 | Abcam | ab63818 | AB_1860806 | Macrophage, monocyte, neutrophil |
| SMA | Abcam | ab7817 | AB_262054 | Smooth muscle |
| STAT5 | Abcam | ab194898 | AB_2924353 | Proliferation, Apoptosis, inflammation |
| Sumo2+3 | Abcam | ab109196 | AB_10859755 | Sumoylation |
| TE-7 | Millipore | CBL271 | AB_93449 | Fibroblasts |
| TH | Cell Signaling Technology | 58844 | AB_2744555 | Nerve marker: peripheral nerve |
| Vimentin | Cell Signaling Technology | 5741 | AB_10695459 | Mesenchymal cell |

| ISH | Vendor | Catalog # | Target |
| --- | --- | --- | --- |
| ACTA2 | Advanced Cell Diagnostics | 444771-C2 | Myofibroblast formation |
| CCL19 | Advanced Cell Diagnostics | 474361 | Regulatory T cell migration |
| CCL2 | Advanced Cell Diagnostics | 423811 | Inflammatory cytokine |
| CCL7 | Advanced Cell Diagnostics | 425261 | Immunosuppressive cell cytokine |
| CCR1 | Advanced Cell Diagnostics | 401881 | Inflammatory cytokine receptor |
| CD3E | Advanced Cell Diagnostics | 553971-C2 | Pan T cell |
| CD68 | Advanced Cell Diagnostics | 560591-C2 | Macrophage |
| CDKN2A | Advanced Cell Diagnostics | 310181 | Tumor suppressor gene |
| COX5B | Advanced Cell Diagnostics | 428051 | Oxidative phosphorylation |
| CSF1R | Advanced Cell Diagnostics | 310811 | M2 Macrocphages |
| CXCL10 | Advanced Cell Diagnostics | 311851 | Inflammatory chemokine, activate/recruit leukocytes |
| CXCL13 | Advanced Cell Diagnostics | 311321 | Chemoattractant, exhaustion marker |
| CXCL9 | Advanced Cell Diagnostics | 440161 | Inflammatory chemokine, activate/recruit leukocytes |
| CXCR3 | Advanced Cell Diagnostics | 539251 | T Cell cytokine receptor |
| CYBB | Advanced Cell Diagnostics | 459371 | phagocytosis |
| GDF15 | Advanced Cell Diagnostics | 600301 | Tumor immunosuppresion |
| GZMB | Advanced Cell Diagnostics | 445971 | Cytotoxic T-cell subset; NK cells |
| IDO1 | Advanced Cell Diagnostics | 602681 | Tumor immunosuppresion |
| IFNB1 | Advanced Cell Diagnostics | 417071 | Type I interferon response |
| IFNG | Advanced Cell Diagnostics | 310501 | T cell activation |
| IL2 | Advanced Cell Diagnostics | 402041 | T cell activation |
| IL32 | Advanced Cell Diagnostics | 541431-C2 | Inflammatory cytokine |
| ISG15 | Advanced Cell Diagnostics | 467741 | Interferon response |
| ITGAX | Advanced Cell Diagnostics | 419151-C2 | Dendritic cells |
| LAG3 | Advanced Cell Diagnostics | 553931 | T cell checkpoints |
| LAMC2 | Advanced Cell Diagnostics | 501371 | Tumor microenvironment |
| LCN2 | Advanced Cell Diagnostics | 559441 | Neutrophils |
| LDHB | Advanced Cell Diagnostics | 531271 | Tumor microenvironment |
| LRRC32 | Advanced Cell Diagnostics | 457631 | TGF-β pathway |
| MARCO | Advanced Cell Diagnostics | 512231 | Activation marker on macrophages |
| MRC1 | Advanced Cell Diagnostics | 564211 | Macrophages |
| MX1 | Advanced Cell Diagnostics | 403831 | Interferon response |
| NCAM | Advanced Cell Diagnostics | 421461-C2 | NK cells |
| PDPN | Advanced Cell Diagnostics | 539751 | Lymphatics |
| PRF1 | Advanced Cell Diagnostics | 407381 | Cytotoxic T/NK cells |
| ROR1 | Advanced Cell Diagnostics | 402831 | Tumor marker |
| STAT1 | Advanced Cell Diagnostics | 469861 | Interferon response |
| STAT2 | Advanced Cell Diagnostics | 470391 | Interferon response |
| TNFa | Advanced Cell Diagnostics | 310421 | Inflammatory cytokine |
| TNFRSF18 | Advanced Cell Diagnostics | 415181 | T cell activation |
| TNFSF10 | Advanced Cell Diagnostics | 411841 | Interferon response |
| TP-63 | Advanced Cell Diagnostics | 601891-C2 | HNSCC marker |
| TUBB | Advanced Cell Diagnostics | 588791 | Tumor microenvronment |
